# Salivary cotinine levels on periodontal conditions

**DOI:** 10.6026/973206300211925

**Published:** 2025-07-31

**Authors:** Gayathri Muralidaran, Nagarathna D.V, Arpita Paul, Ramesh Babu

**Affiliations:** 1 Department of Periodontics, Mamata Dental College, Khammam, Telangana, India; 2 Department of Periodontics, AJ Institute of Dental Sciences, Mangalore, Karnataka, India

**Keywords:** Enzyme linked immunoassay (ELISA), periodontium, smoking, salivary cotinine, tobacco

## Abstract

The periodontal health among beedi smokers and tobacco chewers and its correlation to salivary cotinine levels is of interest. Hence, 60 tobacco-consuming male patients of which 30 patients were beedi smokers and 30 were smokeless tobacco users were enrolled in the study. The gingival health and periodontal health were determined. Unstimulated saliva was collected and subjected to quantitative Enzyme linked immunoassay (ELISA). There was a statistically significant effect on number and duration of beedi smoked than chewed (p<0.001). Beedi smoking has more adverse effects on periodontium than tobacco chewing.

## Background:

Periodontitis is a multifactorial chronic condition where the dental biofilm is found in microcolonies either supra or sub gingivally, including various risk factors like; systemic factors (diabetes, hypertension and immunocompromised conditions like HIV), environmental factors (genetics, tobacco consumption and drug intake for various diseases) and iatrogenic factors defective restorations or prosthesis, improper infection control [1]. Experimental and epidemiologic evidence staunchly reveal that diabetes mellitus and tobacco intake are substantial threats to the occurrence of periodontitis [2, 3]. Evidence supports that periodontal attachment loss, bone loss and pocket depth are more prominent in smokers as opposed to non-smokers [4]. The severity of periodontal diseases and progression rate are correlated with smoking might be due to host interaction, periodontal microbiota and tobacco consumption [5]. India is the second-largest manufacturer and the third-largest consumer of tobacco worldwide [6]. According to the Global Adult Tobacco Survey-2, (GATS 2), every fifth adult in urban India and every third adult in rural India use tobacco in some form or the other [7]. Beedi smoking is the greatest smoking problem in India, along with oral form of tobacco chewing [8]. Cigarette smoke is a spectrum of over 4000 notorious components including carcinogens, carbon monoxide, carbon dioxide, polycyclic aromatic hydrocarbons, sulfur oxides, metals like cadmium and lead, aldehydes, acidic gases, benzene, toluene, formaldehyde, reactive oxidizing radicals, hydrogen cyanide and the chief addictive and psychoactive molecule - nicotine [7]. Primary metabolites of nicotine metabolised in the liver are nicotine glucuronide (3-5%), 2'-hydroxynicotine (1-2%), nicotine N'-oxide (4-7%), nornicotine (0.4-0.8%), cotinine (~75%), nicotine isomethonium ion (0.4-1%). Quantitatively, the most important metabolite formed by cytochrome P450 mediated C-oxidation of nicotine with 70-80% conversion in most mammalian species is the lactam derivative, cotinine at a clearance average of about 45 ml/min [9].

Cotinine binds to activate and desensitize neuronal nicotinic acetychoilne receptors [10]. The properties of cotinine include longer duration of retention in the body (half-life-16hrs), specificity and evident concentration extent thus making it an analyte of preference for quantifying tobacco smoke exposure [11]. Evidence indicates a correlation between chronic periodontitis and cotinine levels in body fluids with smokers. Self- assessed Questionnaires which are commonly followed are inaccurate about the quantity of intake of tobacco and are usually biased [5]. Till date there are no studies assessing the effects of beedi smokers and tobacco chewers who are more prominent form of tobacco use in developing countries like India, on periodontal condition; studies have concentrated more on cigarette form of smoking. Therefore, it is of interest to report the most prominent form of tobacco use that is beedi smoking and tobacco chewing on periodontal condition by estimating cotinine using ELISA.

## Materials and Methods:

The patients aged 25-50 years, visiting the outpatient department of A.J. Institute of Dental Sciences, Mangalore during the year 2015-2017, were examined for the investigation, based on the personal history of habit of smoking these patients were divided into patients who smoke beedi and patients who use tobacco in chewing form. Of all the patients examined according to statistical power for the p value of <0.05 30 patients were enrolled for each group. Out of 60 patients based on inclusion and exclusion criteria 30 patients were beedi smokers (Group B) and 30 patients were smokeless form of tobacco users (Group T). The inclusion criteria were patients between 25 to 50 years of age, patients who were systemically healthy and patients who were currently smoking beedi and chewing any type of tobacco.

## Exclusion criteria:

Persons with systemic ailments predisposing to periodontitis, participants who smoked cigarettes and snuff dippers, subjects who had undergone professional dental prophylaxis within the last 6 months, patients who used antimicrobial mouth rinse in the last 3 months, patients with necrotizing ulcerative periodontitis and aggressive periodontitis, former tobacco users and patients who consume alcohol.

## Ethics and consent to participate:

Ethical approval was acquired from the A.J Ethics Committee, A.J Institute of Medical Sciences and Research Center (IEC Number: AJEC/Rev/66/2014-2015). Written informed consent was obtained from every patient after they had been informed about the study's objective. A single examiner carried out the study throughout the study period and followed the consort guidelines.

## Clinical procedure:

A printed form was utilized to obtain the participants' demographic data assessing age, dental hygiene habits and a detailed history of the various forms of tobacco use, amount per day and number of days. The intra-oral examination was also recorded in the form.

## Collection of saliva:

Sample collection was performed at the standardized time in the morning based on the diurnal rhythm. Participants were required to abstain from eating or rinsing within 60 minutes before collecting the sample. Whole saliva was obtained by drooling into a sterile vial with the forward-leaning head or by letting the saliva pool in the mouth and then spit into the vial. This was placed in aliquots at -20°C until further analysis was done using enzyme linked immunosorbent assay kit (ELISA).

## Intra-oral examination:

Complete periodontal examination included clinical attachment level and probing pocket depth which was recorded using mouth mirror and Williams graduated periodontal probe upto 10 mm with 4 and 6 missing. The indices for the study included the sulcus bleeding index given by Muhlemann and Son (1971), the plaque index given by Silness and Loe (1964) and the gingival index given by Loe and Silness (1963). Clinical attachment loss was assessed at six sites per tooth as the distance between the cementoenamel junction and the base of the pocket. Probing depth was recorded as the distance between the free gingival margin and the base of the pocket.

## Elisa principle:

Standards and unknowns are added to a 96-well microtiter plate along with rabbit antibodies to cotinine and cotinine linked to horseradish peroxidase (conjugate). The cotinine in standards, unknowns and the conjugate competes for the antibody binding sites. After incubation, unbound components are washed away. Bound conjugate is measured by the reaction of the peroxidase enzyme on the substrate tetramethylbenzidine (TMB). This reaction produces a blue color. A yellow color is formed after stopping the reaction with 2- molar sulfuric acid. Optical density is read on a standard plate reader at 450 nm. The amount of cotinine peroxidase detected is inversely proportional to the amount of cotinine present.

## Saliva processing:

On the day of procedure saliva was thawed completely, vortex and centrifuged at 1500 x g (@3000 rpm) for 15 minutes. The collected saliva is added to the 96 well cotinine salivary immunoassay kits by Biogenuix Medesystems Pvt Ltd (Salimetrics). This is a highly sensitive quantitative ELISA kit which could detect salivary cotinine levels of minimum of 0.15ng/ml.

## Procedure:

20 µl of standards, controls and unknowns was taken into appropriate wells. 20 µl of assay diluent into 2 wells to serves as the zero. Later, non-Specific Binding wells, was done by pipetting 120 µl of assay diluent into 2 wells. The enzyme conjugate 1:300 was diluted by adding 50 µl of the conjugate to the 15 ml of assay diluent prepared. 100 of antiserum into all wells, except the nonspecific binding wells (if used), using a multichannel pipette and then the plate was covered with a plate cover. Incubated the plate on a microplate incubator/shaker for 1.5 hours at 37°C with constant mixing at 500-600 rpm.The plate was washed 4 times with 1X wash buffer by pipetting 300 ml of wash buffer into each well and then flipping the liquid into a sink . 200 µl of TMB solution was added to each well using a multichannel pipette. Then mixed at 500 rpm for 5 minutes (or tap to mix) and incubate in the dark for an additional 25 minutes at room temperature. 50 µl of stop solution was added using a multichannel pipette. Mixed on a plate rotator at room temperature for 3 minutes at 500 rpm till, the wells have turned yellow. The plate was read in a plate reader at 450 nm and optical density was obtained. The obtained optical density was used to determine the cotinine level. The obtained clinical and biochemical parameters were updated in excel sheet and further sent for statistical analysis.

## Statistical analysis:

Statistical analysis was done by using SPSS (Statistical Package for Social Sciences) version 17, p<0.05 was regarded as statistically significant. Statistical analysis for comparison of beedi smoking and tobacco chewers was done using unpaired Student's't' test. The correlation between the number of times smoked or chewed per day and the number of years to clinical and biochemical parameters was done using Pearson's correlation coefficient.

## Results:

Out of the 60 patients examined, 30 were patients who smoked beedi and 30 were patients who chewed tobacco. The clinical parameters were evaluated by assessing various indices which include gingival index (GI), plaque index (PI), sulcus bleeding index (SBI), probing pocket depth (PPD) and clinical attachment level (CAL) for both groups. The mean GI was 2.0 in beedi smokers and 1.4 in tobacco chewers. PI recorded showed a mean of 2.1 in beedi smokers and 1.6 in tobacco chewers. GI and PI were significantly higher (p< 0.001) in beedi smokers suggesting poor maintenance of their oral hygiene (Figure 1 - see PDF). SBI had no statistical significance in both the groups (p=0.9) that vasoconstriction is prominent among nicotine users (Figure 2 - see PDF). PPD was statistically significant (p=0.03) with a mean of 6.5 and 5.3 in beedi smokers and tobacco chewers respectively (Figure 3 - see PDF). CAL showed no difference between beedi smokers and tobacco chewers (p=0.4). Pocket depth increased in beedi smokers. Salivary cotinine levels showed no statistically significant difference between both groups (p=0.41). The mean for tobacco chewers (mean=99.5ng/dl) and for beedi smokers (mean= 94.8ng/dl) were at the same level which stated that cotinine is a reliable biomarker for any form of tobacco (Figure 4 - see PDF). Table 1 (see PDF) & 2 (see PDF) shows the correlation which was evaluated between numbers of beedi smoked per day and number of years it has been smoked to clinico-biochemical parameters showed an overall positive correlation to GI, PPD and salivary cotinine levels. A negative impact on number of beedis smoked to PI and SBI enhanced the fact that there would be decrease in bleeding in smokers. This states that inflammation is enhanced effect on gingival and periodontal health. In the case of tobacco chewers, when same parameters were considered, it was noted to have more of a negative impact on GI and PPD, whereas a positive impact on parameters like SBI, PI and salivary cotinine levels. This states that inflammation is enhanced in any form of tobacco on gingival and periodontal health. Beedi smoking has a higher impact on hygiene maintenance, periodontal health when compared to tobacco chewers.

## Discussion:

Tobacco use and its association with oral disease is a major contributor to the global oral disease burden [12, 13]. Tobacco is mainly used either in a smoking form or a smokeless form. Periodontitis is chiefly regarded to be a slowly advancing condition. However, in the presence of environmental and systemic elements like diabetes it enhances the progression of the disease process. In India, tobacco is consumed as beedis (34%), snuff (2%), cigarettes (30%), hookah (9%), chewing tobacco (19%), cigars and cheroots (5%) [14]. The association between periodontitis and smokeless tobacco has also been the topic of meticulous investigations. The interconnection between oral cancer and tobacco consumption is well-established in the literature. However, very few studies present the relation to periodontal health. The present study aimed to compare the cotinine levels in beedi smokers and users of smokeless form of tobacco, as well as to evaluate the influence of different forms of tobacco on periodontal health. Beedi smoking is a widespread mode of tobacco consumption in Southern parts of Asia, considering to nearly one-third of the tobacco yielded in India for smoking [15]. Beedis are manufactured by rolling a rectangular, dried piece of tendu leaf with 0.15-0.25 g of sundried, flaked tobacco. These tobacco-filled leaves deliver more tar, carbon monoxide and nicotine and bear an increased potential of oral cancers [16, 17-18]. The other form that is more prevalent is smokeless tobacco which is employed to elucidate tobacco that is taken without heating or burning while being consumed. The smokeless forms of tobacco are predominant in India and include various forms like mishri, betel quid chewing, khaini, paan, gutka and snuff [19]. A universally utilized biochemical approach to access tobacco smoke exposure is cotinine, a proximate metabolite of nicotine. It can be determined in saliva, blood, or urine and is regarded as a precise estimate of smoking. Cytochrome is caused by cytochrome P450-mediated C-oxidation of nicotine. This product is very stable and has a prolonged half-life on average of 16 hours and is perpetually constant as compared to measuring nicotine directly. Cotinine is detectable in the body fluids of active and passive smokers. Cotinine can be measured by Gas Chromatography (GC), Radioimmunoassay (RIA) or Liquid Chromatography (LC) in various body constituents, including urine, blood, hair, saliva, amniotic fluid and cervical mucus [20]. Asha *et al.* concluded that estimation of salivary cotinine by immunochromatographic assay is an effective means for observing nicotine addiction in tobacco consumers [21]. Bernert *et al.* stated that the serum cotinine levels were very closely associated with cotinine in unstimulated saliva (> by 4%) than to stimulated saliva (> by 41%). Thus, unstimulated saliva is a more dependable biochemical marker [22].

Beedis are an alternate type of cigarette, with higher nicotine content [23]. Beedi smoking had a positive impact on gingival health and this condition further declined on long-term use. This finding is in accordance with the description by Newburn *et al.* [24]. Gingival health assessed by gingival index has a negative impact on tobacco chewing per day and for number of years chewed. This may be attributed to the report that salivary IgA levels are higher in tobacco chewers compared to smokers in unstimulated whole saliva thus providing a protective effect on gingival tissue [25]. Longer duration of smoking (years) showed a significant correlation with plaque index according to Al-Bayaty *et al.* [26] studies. The present study also demonstrated that the plaque index increased over the years due to tobacco chewing, leading to the accumulation of supragingival and subgingival calculus on the tooth surface. Lower gingival bleeding in smokers and tobacco chewers can be attributed to the presence of nicotine, which induces peripheral blood vessel vasoconstriction [27] which was also noted in the present study. The present study also found that bleeding is suppressed after long-term use of beedis (mean 0.35, r=0.02). This aligns with the analysis suggesting that prolonged smoking over the years masks the effect on gingival bleeding during slight probing [26]. Generally, the rationale behind the increased CAL and PPD in patients subjected to tobacco smoke is double. According to the literature, there is no direct alteration of bacterial flora between non-smokers and smokers [28]. Hence, the harmful outcomes of tobacco on the host manifest through dual channels. First, it systemically alters the immune response. Second, it exerts local effects by releasing vasoactive substances and cytotoxic metabolites produced during tobacco combustion, which subsequently affect the vascular responses and fibroblasts [29]. The negative correlation of PPD and CAL in tobacco chewers can be attributed to a systematic review which stated that smokeless tobacco had higher levels of PGE2 and lymphocyte (B and T) and thus may have an initial protective mechanism as the tobacco is chewed [30]. The composition of tobacco chewed might also have an impact on periodontium. Tobacco is chewed mostly with areca nuts. Studies have stated that betel/areca nut chewing had a shielding effect on tooth loss (TL) and clinical attachment loss (CAL) [31]. The true effect of nicotine might be reduced.

Another explanation is that slaked lime, areca nut and smokeless tobacco surge the formation of reactive oxygen species by interacting with the periodontal tissues. This escalates alveolar bone loss and inflammation by reducing endothelial nitric oxide synthase expression and forming pro-inflammatory cytokines as established by Javed *et al.* in 2008 [32]. A study done for urinary cotinine level detection in beedi smokers and tobacco chewers found that cotinine is more in tobacco chewers, whereas in this study it was similar in both the groups. Mc Gurie *et al.* estimated the salivary cotinine using high performance liquid chromatography and found higher concentrations both in GCF and saliva [33]. According to Behera *et al.* an optimal salivary or plasma cotinine cut-point of 15ng ml-1 was decided to differentiate non-smokers from smokers [34]. The difference between beedi smoking and tobacco chewing can also be accredited to the truth that the nicotine level varies between each beedi smoked and the amount of tobacco chewed. Several factors play a role in the quantity of cotinine being estimated. For beedi smokers' consumption of nicotine during smoking relies on puff volume, the extent of dilution with room air, depth of inhalation, intensity and rate of puffing. In tobacco chewers, the amount of tobacco taken each time, the duration for which it is placed in the mouth and the extent of dilution with other products like slaked lime or areca nut can influence the gingival and periodontal health. In general, the cotinine concentration also depends on the time when the saliva was collected after the last smoke or chewed and on the salivary flow rate. These parameters can also influence the results but couldn't be taken into consideration as it was not practicable. However, tobacco chewers had a higher gingival and plaque index compared to gingivally healthy subjects. Tobacco chewing also impacted the periodontium, with elevated clinical attachment loss and probing pocket depth, relative to its lesser sulcus bleeding index. Surya *et al.* [35] concluded that half of the subjects who chewed tobacco had periodontal destruction and 66% had a loss of attachment indicating deteriorated oral hygiene, gingival health and periodontal status. This findings correlate with the study by Giri *et al.* [36] who concluded that all of their study subjects exhibited poor oral hygiene and increased periodontal destruction. A study done by Praveen *et al.* in the year 2017 showed a strong association between salivary cotinine levels and severity of periodontitis [37]. This study based on the obtained clinic-biochemical parameters can be inferred that both beedi smokers and tobacco chewers has adverse effect on gingival and periodontal health. Beedi has more adverse effect on clinical parameters when compared to tobacco chewers. Salivary cotinine is a reliable biomarker for any form of tobacco consumption. The salivary cotinine levels obtained were slightly higher in tobacco chewers. Number of beedi smoked or tobacco chewed per day and number of years it's been consumed also has effect on the clinico-biochemical parameters. A large-scale study should be carried out to obtain a correlation to the number of beedi smoked or chewed to clinico-biochemical parameters. A detailed study is yet to be done regarding the pathway of destruction of periodontium by beedi smoking and tobacco chewing at a molecular level.

## Conclusion:

It is inferred that beedi consumption poses harmful effects for a longer duration when compared to tobacco chewers. Beedi has more adverse effects on gingival and periodontal health when compared to tobacco chewers. Nevertheless, the salivary cotinine was found to be similar in tobacco chewers than beedi smokers. Although beedi has a higher impact on periodontal tissue, tobacco chewing also causes significant damage. Therefore, tobacco consumption in any form has a detrimental effect on periodontal tissues. Further, salivary cotinine serves as a reliable biochemical marker for assessing tobacco use in all forms.

## Clinical relevance:

Beedi smoking poses a significant public health challenge in India and contributes substantially to the global tobacco epidemic. Additionally, the widespread use of smokeless tobacco products orally exacerbates the issue, further compounding the public health concerns related to tobacco use. A commonly employed biochemical method of gauging tobacco smoke exposure is through the measurement of cotinine, a metabolic by product of nicotine. It can be determined in saliva, blood, or urine and is regarded as a precise estimate of smoking.

## Added value:

In the current study, the analysis of clinic-biochemical parameters suggests that both beedi smokers and tobacco chewers harm the health of the gingiva and periodontium. Notably, beedi smoking appears to have a more pronounced adverse effect on certain clinical parameters when compared to tobacco chewing.

## Clinical implication:

Salivary cotinine, a metabolite of nicotine, is a widely recognized and dependable biomarker used for the detection of various forms of tobacco consumption.

## Presentation(s) or Awards at a meeting:

None

## Source(s) of Support and Funding:

No funding was obtained for this study
